# Back-propagation-assisted inverse design of structured light fields for given profiles of optical force

**DOI:** 10.1515/nanoph-2023-0101

**Published:** 2023-05-01

**Authors:** Xiaoshu Zhao, Haoze Lin, Huajin Chen, Hongxia Zheng, Jack Ng

**Affiliations:** State Key Laboratory of Surface Physics and Department of Physics, Fudan University, Shanghai 200433, China; Department of Computer Science, Columbia University, New York, NY 10027, USA; School of Electronic Engineering, Guangxi University of Science and Technology, Liuzhou, Guangxi 545006, China; Guangxi Colleges and Universities Key Laboratory of Microwave Communication and Micro-Nano Photoelectric Technology, Liuzhou, Guangxi 545006, China; Department of Physics, Southern University of Science and Technology, Shenzhen, Guangdong 518055, China

**Keywords:** back-propagation algorithm, inverse design, optical force, structured light field

## Abstract

Designing a monochromatic spatially-structured light field that recovers the pre-specified profile of optical force (OF) exerted on a particle is an inverse problem. It usually requires high dimensional optimization and involves lengthy calculations, thus remaining little studied despite decades of research on OF. We report here the first attempt to attack this inverse design problem. The modus operandi relies on the back-propagation algorithm, which is facilitated by the currently available machine learning framework, and, in particular, by an exact and efficient expression of OF that shows only polynomial and trigonometric functional dependence on the engineered parameters governing the structured light field. Two illustrative examples are presented in which the inversely designed structured light fields reproduce, respectively, a predefined spatial pattern of OF and a negative longitudinal OF in a transversely trapping area.

## 1 Introduction

Optical forces (OFs) exerted on an object illuminated by light have been extensively explored and exploited for intact manipulation of microparticles since the pioneering work of Ashkin in 1970s [1]. Despite decades of research [1–31] in many scientific fields ranging from optics to chemistry and biological science, the inverse design of monochromatic optical fields based on the pre-specified OF remains little explored, in particular, beyond the dipole limit. The earliest implementations of inverse design of some desired light patterns include finding holograms for optical trapping [32]. Later on, the technique was incorporated into commercial holographic trapping instruments as well as the design of tractor beams [33], but all limited to the Rayleigh (dipole) approximation. The inverse design of light fields capable of projecting a pre-specified profile of OF on particle of arbitrary size, however, remains challenging, due to the notoriously complex relationship between the OF and the parameters defining the light field, which impedes the optimization. This paper reports the first attempt to attack this problem.

Given a reasonably predefined spatial pattern of OF, in order to inversely design a structured optical field that can produce the desired pattern of OF on a micro-particle immersed in it, there are three issues to be solved.

The first issue is how to depict a general monochromatic optical field. Theoretically, a generic optical field can be described by its electric field of the form, see, e.g., [34] for a proof,

(1) $$\begin{array}{c} E=\underset{4\pi }{\oint }e_{u}\;{\text{e}}^{\text{i}ku\cdot r}\;\;\text{d}\Omega_{u}, \end{array}$$

where ***u*** is the real unit vector denoting the direction of the wave vector ***k***, and ***e***_***u***_, depending on ***u*** but not on ***r***, is perpendicular to ***u***. The integration is over the unit sphere of the directions of wave vectors ***k*** = *k* ***u***. The time dependence e^−i*ωt*^ is assumed. Numerically, Eq. (1) suggests that a generic field can be cast into a summation of a myriad of plane waves

(2) $$E=\overset{n_{p}}{\underset{j=1}{\sum }}E_{j}\;{\text{e}}^{\text{i}k_{j}\cdot r}\;\text{with}\;E_{j}=E_{0}\left(p_{j}{\hat{\theta }}_{k_{j}}+q_{j}{\hat{\varphi }}_{k_{j}}\right),$$

where *n*_*p*_ is the number of the constituent plane waves, ***E***_*j*_ is the complex amplitude vector, $k_{j}=k{\hat{k}}_{j}$ is the real wave vector for the *j*th plane wave. Two complex numbers *p*_*j*_ and *q*_*j*_ characterize the amplitude, polarization, and phase at the position vector ***r*** = 0. The unit vectors ${\hat{\theta }}_{k_{j}}$ and ${\hat{\varphi }}_{k_{j}}$ denote, respectively, the directions of increasing polar angle and azimuthal angle in the spherical coordinate system for the *j*th wave vector ***k***_*j*_ so that ${\hat{k}}_{j}$ , ${\hat{\theta }}_{k_{j}}$ , and ${\hat{\varphi }}_{k_{j}}$ form a right-handed triplet of unit vectors. With Eq. (2), one is, in principle, ready to recover arbitrary spatially-structured light [35, 36] by tuning the number *n*_*p*_ of the constituent plane waves, and the polar angle *θ*_*j*_ and azimuthal angle *φ*_*j*_ of ***k***_*j*_, as well as the two complex numbers *p*_*j*_ and *q*_*j*_ for each constituent plane wave. Given all these parameters, with the use of spatial light modulators, most theoretically designed structured light fields can be realized experimentally, with the idea of angular spectrum representation [37].

The second issue is the calculation of the OF exerted on a particle illuminated by the structured light field given by Eq. (2). The inverse design usually involves extensive calculations [38–40], the “brute-force” calculation is obviously not feasible. The generalized Lorenz-Mie theory (GLMT) [41] works well for calculating OF on spherical particles, see, e.g., [42, 43]. However, for the structured light field in Eq. (2) that is composed of multiple interferential plane waves, the GLMT requires expanding each plane wave in a series of vector spherical wave functions [44, 45] to obtain the beam shape coefficients [41]. Although this can be worked out analytically, see, e.g., [41–44] it would require computing the associated Legendre functions and their derivatives. This impedes the analytical calculation of the gradient of loss (see, below) with respect to the engineered parameters with standard back-propagation [46] by direct use of the common-used machine learning frameworks [47, 48]. In this paper we adopt our recently-developed approach [34, 49], [50], [51], [52], which calculates the OF exerted on a particle immersed in an arbitrary number of interferential plane waves without resorting to the vector spherical wave functions. The approach is further reformulated in such a way that the expression of the OF involves only the polynomials and trigonometric functions. So it fully facilitated the application of the back-propagation within the currently available machine learning frameworks, not having to resort to neural networks (NNs) for approximating the OF calculation. For comparison, we have trained a NN to approximate the OF with high degrees of precision. The trained NN generates the results in time less by around 50 % compared with our reformulated approach. Nevertheless, it takes much time in training, and, to be worse, needs to be retrained when the size or scattering property of particle is changed. The details of the OF calculation formulation will be given in Section 2.

The third issue concerns the optimization. To this end we design a loss function measuring the difference between the pre-specified OF and the calculated OF with the given engineered parameters chosen from *n*_*p*_, *θ*_*j*_, *φ*_*j*_, *p*_*j*_ and *q*_*j*_, subject to some physical constraints for the easiness of experimental realization. Then with the standard back-propagation, the gradient of the loss function with respect to the engineered parameters are obtained “analytically”. This is achieved by using the reformulated OF expression written in terms of only the polynomials and trigonometric functions while circumventing the much more complicated special functions in the framework of GLMT. Next, the ADAM optimizer [53, 54] is used to optimize the engineered parameters for decreasing the loss function. This is somewhat similar to the optimization process where the direct search algorithm is used to calculate holograms [55]. After some epoches of optimization, we arrive at a structured light field in the form of Eq. (2) which reproduces the desired spatial pattern of OF to a reasonable accuracy. Details of the optimization procedure will be presented in Section 3 by two illustrative examples.

## 2 Formulation

In this section, the Cartesian multipole expansion theory [34, 49], [50], [51], [52] is first recapitulated for the calculation of the OF on a spherical particle. Then we reformulate the OF expression to a form that show only the polynomial and trigonometric functional dependence on the engineered parameters *p*_*j*_, *q*_*j*_, *θ*_*j*_ and *φ*_*j*_. This is done for the purpose of the calculation of gradient of the loss function by direct use of the back-propagation within the current machine learning framework, avoiding the tedious mathematical calculations involving the associated Legendre functions and their derivatives in the framework of GLMT. For simplicity, we set *E*_0_ = *k* = *ɛ*_0_ = *μ*_0_ = *c* = 1 during derivation. The final expression for OF is thus in unit of $\varepsilon_{0}\varepsilon_{b}E_{0}^{2}/k^{2}$ , where *ɛ*_*b*_ denotes the relatvie electric permittivity in the background medium.

According to the Cartesian multipole expansion theory [34, 49], [50], [51], the time-averaged OF ⟨ ***F*** ⟩ on a spherical particle illuminated by a generic structured light field given by Eq. (2) can be written as

(3) $$\begin{array}{cc} \left\langle F\right\rangle & =\underset{l}{\sum }\left[\left\langle F_{\text{int}}^{\text{e}\left(l\right)}\right\rangle +\left\langle F_{\text{int}}^{\text{m}\left(l\right)}\right\rangle +\left\langle F_{\text{rec}}^{\text{e}\left(l\right)}\right\rangle \right.\left.+\left\langle F_{\text{rec}}^{\text{m}\left(l\right)}\right\rangle +\left\langle F_{\text{rec}}^{\text{x}\left(l\right)}\right\rangle \right],\\ \left\langle F_{\text{int}}^{\text{e}\left(l\right)}\right\rangle & =-\frac{\pi \left(2l+1\right)}{l\left(l+1\right)}\;\text{Im}\underset{i,j}{\sum }a_{l}\left[Q_{l,\;ij}^{\left(1\right)}Z_{\text{ee},ij}^{\left(1\right)}\right.\left.-Q_{l,ij}^{\left(2\right)}Z_{\text{mm},ij}^{\left(1\right)}\right],\\ \left\langle F_{\text{int}}^{\text{m}\left(l\right)}\right\rangle & =-\frac{\pi \left(2l+1\right)}{l\left(l+1\right)}\;\text{Im}\underset{i,j}{\sum }b_{l}\left[Q_{l,ij}^{\left(1\right)}Z_{\text{mm},ij}^{\left(1\right)}\right.\left.-Q_{l,ij}^{\left(2\right)}Z_{\text{ee},ij}^{\left(1\right)}\right],\\ \left\langle F_{\text{rec}}^{\text{e}\left(l\right)}\right\rangle & =-\frac{\pi }{2{\left(l+1\right)}^{2}}\;\text{Im}\underset{i,\;j}{\sum }a_{l+1}a_{l}^{*}\left[R_{l,\;ij}^{\left(1\right)}Z_{\text{ee},\;ij}^{\left(1\right)\;*}\right.\\ & \;\left.-R_{l,\;ij}^{\left(2\right)}Z_{\text{mm},\;ij}^{\left(1\right)\;*}-4i\;R_{l,\;ij}^{\left(3\right)}S_{\text{em},\;ij}^{\left(1\right)\;*}+R_{l,\;ij}^{\left(4\right)}Z_{\text{ee},\;ij}^{\left(1\right)}\right.\\ & \;\left.-R_{l,\;ij}^{\left(5\right)}Z_{\text{mm},\;ij}^{\left(1\right)}+4i\;R_{l,\;ij}^{\left(6\right)}S_{\text{em},\;ij}^{\left(1\right)}\right],\\ \left\langle F_{\text{rec}}^{\text{m}\left(l\right)}\right\rangle & =-\frac{\pi }{2{\left(l+1\right)}^{2}}\;\text{Im}\underset{i,\;j}{\sum }b_{l+1}b_{l}^{*}\;\left[R_{l,\;ij}^{\left(1\right)}Z_{\text{mm},\;ij}^{\left(1\right)\;*}\right.\\ & \;\left.-R_{l,\;ij}^{\left(2\right)}Z_{\text{ee},\;ij}^{\left(1\right)\;*}-4i\;R_{l,\;ij}^{\left(3\right)}S_{\text{em},\;ij}^{\left(1\right)}+R_{l,\;ij}^{\left(4\right)}Z_{\text{mm},\;ij}^{\left(1\right)}\right.\\ & \;\left.-R_{l,\;ij}^{\left(5\right)}Z_{\text{ee},\;ij}^{\left(1\right)}+4i\;R_{l,\;ij}^{\left(6\right)}S_{\text{em},\;ij}^{\left(1\right)\;*}\right],\\ \left\langle F_{\text{rec}}^{\text{x}\left(l\right)}\right\rangle & =-\frac{\pi \left(2l+1\right)}{2l^{2}{\left(l+1\right)}^{2}}\;\text{Im}\underset{i,\;j}{\sum }a_{l}b_{l}^{*}\left[R_{l,\;ij}^{\left(4\right)}\left(Z_{\text{mm},\;ij}^{\left(1\right)}\right.\right.\left.-Z_{\text{ee},\;ij}^{\left(1\right)\;*}\right)\\ & \;\left.+R_{l,\;ij}^{\left(5\right)}\left(Z_{\text{mm},\;ij}^{\left(1\right)\;*}-Z_{\text{ee},\;ij}^{\left(1\right)}\right)\right.\\ & \;\left.+4iR_{l,\;ij}^{\left(6\right)}S_{\text{em},\;ij}^{\left(1\right)\;*}+4iR_{l,\;ij}^{\left(7\right)}S_{\text{em},\;ij}^{\left(1\right)}\right]. \end{array}$$

where the superscript * denotes the complex conjugate, *a*_*l*_ and *b*_*l*_ are the Mie coefficients [56], $Q_{l,ij}^{\left(n\right)}$ with *n* = 1, 2 and $R_{l,ij}^{\left(n\right)}$ with *n* = 1, 2, …, 7 are polynomials of $x_{ij}={\hat{k}}_{i}\cdot {\hat{k}}_{j}$ given by Eq. (15) in Supplementary Information, and the vector quantities read

(4) $$\begin{array}{cc} S_{\text{em},ij}^{\left(1\right)} & =E_{i}\times B_{j}^{*}\;{\text{e}}^{\text{i}\;\left(k_{i}-k_{j}\right)\cdot r},\\ Z_{\text{ee},ij}^{\left(1\right)} & =G_{\text{ee},ij}^{\left(1\right)}-i\;S_{\text{em},ij}^{\left(1\right)},\\ Z_{\text{mm},ij}^{\left(1\right)} & =G_{\text{mm},ij}^{\left(1\right)}-i\;S_{\text{em},ij}^{\left(1\right)*},\\ G_{\text{ee},ij}^{\left(1\right)} & =-i\;\left(k_{j}\cdot E_{i}\right)E_{j}^{*}\;{\text{e}}^{\text{i}\;\left(k_{i}-k_{j}\right)\cdot r},\\ G_{\text{mm},ij}^{\left(1\right)} & =-i\;\left(k_{i}\cdot B_{i}\right)B_{j}^{*}\;{\text{e}}^{\text{i}\;\left(k_{i}-k_{j}\right)\cdot r}, \end{array}$$

with ***E***_*i*_ and ***B***_*i*_ denoting, respectively, the complex amplitude vectors for the electric and magnetic fields of the *i*th plane wave.

Equations (3) and (4) represent an alternative approach for the forward calculation of the OF exerted on arbitrarily-sized spherical particle immersed in any given structured light described by Eq. (2). The formulation, which is based on the Cartesian multipole expansion, does not need to expand each plane wave in Eq. (2) in a series of the vector spherical wave functions as in the spherical multipole-based formulation [41], [42], [43, 57], [58], [59]. It therefore leads to a greatly enhanced computational speed that is even comparable to a trained NN approximation. For the purpose of inverse design, we reformulate the OF expressions in a form that further facilitates the calculation of the gradient of loss function with respect to the engineered parameters based on the standard back-propagation [46]. The reformulated OF expression read, after lengthy algebra,

(5) $$〈F〉=\text{Re}\underset{i,j}{\sum }〈F_{ij}〉{\text{e}}^{\text{i}\;\left(k_{i}-k_{j}\right)\cdot r},$$

where ⟨ ***F***_*ij*_⟩ are ***r*** independent, and its Cartesian components are worked out to be quadratic form in terms of polarization parameters *p* and *q*,

(6) $$\begin{array}{cc} F_{x,ij} & =N_{ij}^{\left(1\right)}p_{i}p_{j}^{*}+N_{ij}^{\left(2\right)}q_{i}q_{j}^{*}+N_{ij}^{\left(3\right)}p_{i}q_{j}^{*}+N_{ij}^{\left(4\right)}q_{i}p_{j}^{*},\\ F_{y,ij} & =N_{ij}^{\left(5\right)}p_{i}p_{j}^{*}+N_{ij}^{\left(6\right)}q_{i}q_{j}^{*}+N_{ij}^{\left(7\right)}p_{i}q_{j}^{*}+N_{ij}^{\left(8\right)}q_{i}p_{j}^{*},\\ F_{z,ij} & =N_{ij}^{\left(9\right)}p_{i}p_{j}^{*}+N_{ij}^{\left(10\right)}q_{i}q_{j}^{*}+N_{ij}^{\left(11\right)}p_{i}q_{j}^{*}+N_{ij}^{\left(12\right)}q_{i}p_{j}^{*}. \end{array}$$

The coefficients $N_{ij}^{\left(n\right)}$ , with *n* = 1, 2, …, 12, are products of polynomials of *x*_*ij*_ and the trigonometric functions of *θ*_*i*_, *φ*_*i*_, *θ*_*j*_, and *φ*_*j*_, befitting the back-propagation. Their explicit expressions are given in Supplementary Information by Eq. (16).

With Eqs. (5) and (6), the calculation of OF on a spatial grid of 18,040 points in the next section takes only a fraction of second on a mobile computer with NVIDIA GeForce GTX 1060 (6G), allowing for the expeditious optimization with the gradient descent method.

## 3 Demonstration results and discussion

In this section we present two demonstration examples for the inverse design of structured light fields from the pre-specified OF.

### 3.1 Trapping inside of a prespecified pattern

The first example concerns capturing particle transversely inside of a predefined pattern. The non-magnetic particle to be trapped transversely has relative permittivity *ɛ*_*s*_ = 2.53 and radius *r*_*s*_ = 0.5*λ*_0_ with *λ*_0_ = 532 nm denoting the wavelength of the operating structured light in vacuum. The particle is immersed in water with a refractive index of 1.33. The OF exerted on the particle is decomposed into the longitudinal supporting OF *Y*_*z*_(*x*, *y*) in *z* direction (the particle is not trapped along *z* by the OF), which pushes the particle against, say, the glass plate, and the transverse trapping OF ***Y***_*t*_(*x*, *y*), which confines the particle inside of the predefined pattern of ‘FDU’. A rectangular area with *x* extending from 0 to 40.8*λ* and *y* from 0 to 17.4*λ* is set to be the region for optical manipulation. Here *λ* stands for the wavelength in the background medium (water). The area is divided into a grid of 205 × 88 = 18,040 points, with a resolution (grid size) of 0.2*λ*. Such an area is referred to as training area and the grid points inside referred to as training samples throughout the paper, since we are “training” a structured light field to reproduce a pre-designed pattern of OF.

Initially, the value of the longitudinal OF *Y*_*z*_(*x*, *y*) at *z* = 0 is set to 3 within the pattern ‘FDU’ and 0 otherwise, with its profile displayed in Figure 4(a) in Supplementary Information. To avoid the unphysical sharp change of OF at the edge of the pattern, we apply 8 discrete convolutions (see, e.g., [60]) to the *Y*_*z*_(*x*, *y*) profile and get a smoothed profile with practical physical feasibility. The convolution, padded with a 1 × 1 border of zeros and using 1 × 1 strides, has a kernel of 3 × 3 with all values set to 1/9. The output size of each convolution stays the same as the input size, resulting in a smoothed profile of the longitudinal OF as exhibited in Figure 4(b). Each convolution here is indeed equivalent to averaging the value of longitudinal OF over 3 × 3 = 9 grid points. To confine the particle inside of the ‘FDU’ pattern, the values of the two Cartesian components of the transverse trapping force ***Y***_*t*_(*x*, *y*) at *z* = 0 plane are set to be

(7) $$\begin{array}{c} Y_{x}\left(x,y\right)=6\left[Y_{z}\left(x+0.2\lambda ,y\right)-Y_{z}\left(x-0.2\lambda ,y\right)\right],\\ Y_{y}\left(x,y\right)=6\left[Y_{z}\left(x,y+0.2\lambda \right)-Y_{z}\left(x,y-0.2\lambda \right)\right], \end{array}$$

which has its profile as shown in Figure 4(c).

To realize the OF patterns as shown in Figure 4(b) and (c), we use *n*_*p*_ = 100 plane waves to construct the structured light of the form Eq. (2). To facilitate the implementation using the space light modulator, all the plane waves are supposed to have the electric field polarized parallel to the *x*-*o*-*z* plane.

This is achieved by setting *p*_*i*_ and *q*_*i*_ for the *i*th plane wave to

(8) $$\begin{array}{cc} & p_{i}=\frac{\cos\varphi_{i}}{\sqrt{{\left(\cos\varphi_{i}\right)}^{2}+{\left(\cos\theta_{i}\sin\varphi_{i}\right)}^{2}}}\;A\;{\text{e}}^{\text{i}\gamma_{i}},\\ & q_{i}=-\frac{\cos\theta_{i}\sin\varphi_{i}}{\sqrt{{\left(\cos\varphi_{i}\right)}^{2}+{\left(\cos\theta_{i}\sin\varphi_{i}\right)}^{2}}}\;A\;{\text{e}}^{\text{i}\gamma_{i}}, \end{array}$$

where *θ*_*i*_ and *φ*_*i*_ are, respectively, the polar angle and azimuthal angle of the *i*th wave vector, and *γ*_*i*_ is its phase at *z* = 0. All plane waves are set to have the same amplitude *A*. We optimize the structured light through the change of the engineered parameters *θ*_*i*_, *φ*_*i*_, *γ*_*i*_, and *A* such that the optimized light can reproduce the OF profiles shown in Figure 4(b) and (c). Initially, *A* is set to 0.05, and *φ*_*i*_ and *γ*_*i*_ are randomly distributed in the range of 0 to 2π, while *θ*_*i*_ randomly distributed between 0 and 0.08π. During the optimization, all *θ*_*i*_ are confined to be less than 0.16π. It is noted that given OF as displayed in Figure 4(b) and (c), we actually do not know what the value of *A* should be. So *A* is set to be an engineered parameter, though it does not change the pattern of OF.

The loss function *L* is then set to be the mean square deviation with an *L*2 regularization of *A*, reading

(9) $$L=\frac{1}{N}\sum_{\left(x,y\right)}{\left|a\cdot \left[F\left(x,y\right)-Y\left(x,y\right)\right]\right|}^{2}+\eta A^{2},$$

where *N* denotes the number of training samples. ***Y*** (*x*, *y*) and ***F*** (*x*, *y*) are the designed value (see, Figure 4(b) and (c)) and the calculated value of the OF, respectively. The vector ***a*** is used to adjust the proportion of the tolerance in transverse and longitudinal OFs. η is a hyperparameter for regularization with respect to amplitude. The summation runs over all *N* = 18,040 grid points (training samples) in the training area. In the optimization (training), we set ***a*** = (2.5, 2.5, 1) and η=200 .

**Figure 1:**
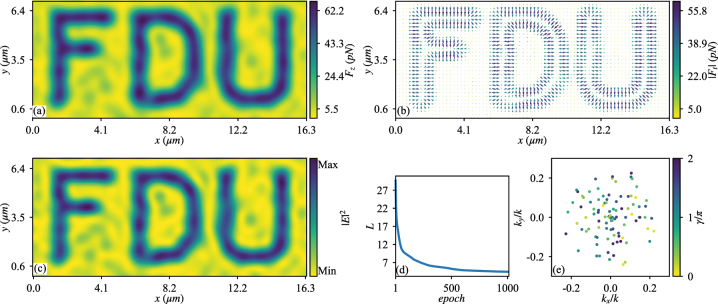
The calculated longitudinal monotonic OF *F*_*z*_ (a) and transverse trapping OF ***F***_*t*_ (b) exerted on a particle with *r*_*s*_ = 0.5*λ*_0_ and *ɛ*_*s*_ = 2.53, placed at *z* = 0 in water and illuminated by the structured light optimized for producing the ‘FDU’ pattern of OF. Each plane wave has *AE*_0_ = 8.68 × 10^5^ V/m. In panel (b) the arrows denote the directions of ***F***_*t*_, while their lengths and colors denote the magnitudes of ***F***_*t*_. (c) The profile of |*E*|^2^ of the optimized structured light in the *x*-*o*-*y* plane. (d) The change of the loss function versus epoch of optimization. (e) The wave vectors and phases of the constituent plane waves, where *k*_*x*_ and *k*_*y*_ of each point denote the transverse Cartesian components of the wave vector, with the color denoting the phase *γ*, see, Eq. (8).

The ADAM optimizer [53, 54] is used to decrease the loss function *L*, with the gradient of *L* with respect to the engineered parameters *θ*_*i*_, *φ*_*i*_, *γ*_*i*_, and *A* obtained analytically with the standard back propagation [46], available in the machine learning framework like Tensorflow [47]. The optimization is completed within a few minutes with learning rate 0.001. The profiles of the longitudinal and transverse OFs exerted on the particle, the light intensity in the *x*-*o*-*y* plane, the loss function *L* versus epoch, and the distribution of the wave vectors for the *n*_*p*_ = 100 plane waves are shown in Figure 1. In Figure 5 in Supplementary Information we also show the gradient (conservative) and scattering (non-conservative) OFs based on the decomposition formulation proposed in [49], [50], [51, 59]. The scattering force dominates the longitudinal supporting OF, while the transverse trapping force stems mainly from the gradient force, with its optical potential shown in Figure 5(e).

The transverse trapping of particle inside of the prespecified pattern is not limited to *z* = 0 plane. Particle can be so captured within the range of −3*λ* ≲ *z* ≲ 3*λ*, as shown in Figure 6 in Supplementary Information, where the results at *z* = ±3*λ* are displayed. In addition, the optimized structured light obtained for particle with *r*_*s*_ = 0.5*λ*_0_ also work quite well for a particle of radius ranging from 0.1*λ*_0_ to 1.0*λ*_0_. A case which yields a structured light field that can confine particle of radius *r*_*s*_ = 1.2*λ*_0_ inside of the ‘FDU’ pattern is also shown in Figure 7 in Supplementary Information.

### 3.2 Optical pulling force

As the second demonstration example, in this subsection, we apply the inverse design algorithm to find some scenarios of optical pulling.

Pulling force against the wave propagation was first theoretically discussed in acoustic wave by Marston [61]. The earliest experimental demonstration of an optical pulling force was reported in [62], where optical pulling is achieved by concurring gradient and scattering forces in solenoid beams. The optical pulling force (negative OF) originated exclusively from the scattering force was first identified in the case of particle illuminated by Bessel beams [12, 13, 22], which can be modeled by a superposition of plane waves with their wave vectors ***k***_*i*_ lying on a conical surface. That means, the *z* components of all wave vectors are the same, giving in principle rise to a non-diffractive optical field where the OF stays unchanged along *z* axis. As a demonstration example, for simplicity, we assume that all wave vectors of the constituent plane waves making up the structured light have an equal polar angle.

We choose to recover a simple case as in [63], where a nonmagnetic particle with a permittivity *ɛ*_*s*_ = 7 is located in vacumm and illuminated by a zero-order transverse magnetic (TM) Bessel beam with cone angle *α* = 70°. The particle size is *kr*_*s*_ = 2.7. We optimize a structured light field consisting of 6 plane waves to achieve the negative longitudinal OF in a transversely trapped area on the same particle. In practice, such a pulling force is expected to be realizable on a particle fabricated by materials such as silicon carbide (SiC) or titanium dioxide (TiO_2_), which have refractive indices 2.65 and 2.95, respectively, at wavelength around 530 nm [64].

As the OF keeps invariable with *z*, we only need to specify the OF in the *x*-*o*-*y* plane. So we first choose a circle with a radius of 0.44*λ*_0_ centered at the origin as the training area, and select training samples evenly in the training area with the resolution of 0.08*λ*_0_. The number of training samples reduces thus to *N* = 97 and the value of the OF ***Y*** (*x*, *y*) is prespecified according to

(10) $$Y\left(x,y\right)=-2.14\hat{\rho }-2\hat{z},$$

where $\hat{\rho }$ and $\hat{z}$ are unit base vectors in the cylindrical coordinate system. Obviously, a particle subject to this OF can be stably trapped transversely near the origin, while being pulled towards light source by the negative longitudinal OF.

Next, we choose the polar angle of all 6 wave vectors to be *θ*_*i*_ = 70° [63], set the azimuthal angle of the *i*th plane wave to *φ*_*i*_ = (*i* − 1)π/3 to retain cylindrical symmetry. The polarization parameters are rewritten as

(11) $$\begin{array}{c} p_{i}=A_{i}\cos\beta_{i}\;{\text{e}}^{\text{i}\gamma_{i}^{\left(1\right)}},\\ q_{i}=A_{i}\sin\beta_{i}\;{\text{e}}^{\text{i}\gamma_{i}^{\left(2\right)}}, \end{array}$$

where *A*_*i*_, *β*_*i*_, $\gamma_{i}^{\left(1\right)}$ , and $\gamma_{i}^{\left(2\right)}$ are engineered parameters, denoting the real amplitude, the polarization and the phase at *z* = 0 of each plane wave, respectively.

Similar to Eq. (9), the loss function *L* is defined by

(12) $$L=\frac{1}{N}\sum_{\left(x,y\right)}{\left|a\cdot \left(F\left(x,y\right)-Y\left(x,y\right)\right)\right|}^{2}+\eta \sum_{i}A_{i}^{2},$$

where η=4 , the summation is over all *N* = 97 training samples, and the hyperparameter ***a*** is chosen to

(13) $$a=\left\{\begin{array}{cc} \left(1,1,10\right),\; & k\sqrt{x^{2}+y^{2}}\leq 0.53,\\ \left(1,1,0\right),\; & \text{otherwise.} \end{array}\right.$$

**Figure 2:**
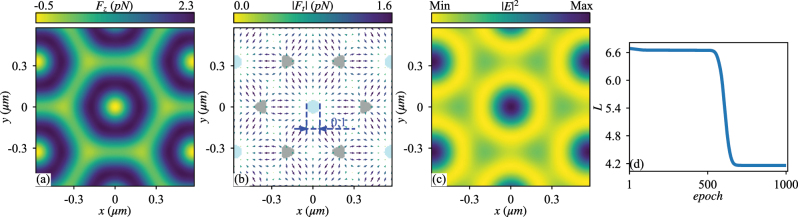
The same as Figure 1 except that the particle is placed in vacuum and has a radius of *r*_*s*_ = 0.43*λ*_0_ and *ɛ*_*s*_ = 7 while the structured light is optimized to produce a transverse trapped area in the central part with a negative longitudinal OF. In panel (b), the shaded parts denote an area of negative longitudinal OF, with the colored ones exhibiting stable transverse trapping. The optimized structured light consists of 6 plane waves, all of which have the magnetic field perpendicular to *z*. The particle is trapped at the intensity maximum basically by the gradient OF.

Again, the ADAM optimizer is used to decrease the loss function *L*. The gradient of *L* with respect to the engineered parameters are obtained again by the back-propagation. The final results turns out to be *q*_*i*_ = 0 with all *p*_*i*_ being equal, corresponding to the TM field [63], in which the magnetic field polarized in a plane normal to *z*. The results indicate that all the 6 constituent plane waves have the same amplitude, phase, and polarization. The profiles of the longitudinal and transverse OFs, light intensity, and change of loss function versus training epoch are shown in Figure 2.

Due to the limited number of constituent plane waves, the structured light, and thus the transverse spatial distribution of OF show obvious periodicity, resulting in a periodic array of trapped area with negative OF. Figure 2(a)–(c) actually exhibit a period in the *x*-*o*-*y* plane. Besides, a comparison between Figure 2(b) and (c) suggests that the trapping originates from the gradient force, as confirmed by the calculated gradient and scattering forces (not shown here). The particle is trapped at the intensity maxima.

Next, let us look at a larger particle with radius *r*_*s*_ = 0.5*λ*_0_ and *ɛ*_*s*_ = 2.53 in vacuum.

To generate a transverse trapped area with longitudinal pulling OF, we still use Eq. (10) for OF and Eq. (12) for loss function, with η=5 and

(14) $$a=\left\{\begin{array}{cc} \left(1,1,20\right),\; & k\sqrt{x^{2}+y^{2}}\leq 1.3,\\ \left(1,1,0\right),\; & \text{otherwise.} \end{array}\right.$$

The radius of the training area is increased to 0.84*λ*_0_. The resolution (grid size) keeps to be 0.08*λ*_0_ and the number of training samples (grid points that are included in the calculation of loss) becomes *N* = 349. The optimization becomes more difficult as the particle gets larger. Therefore, we increase the number of plane waves that make up the structured light to *n*_*p*_ = 10 and all polar angle to *θ* = 0.44π (79.2°). The azimuthal angle of the *i*th plane wave is set to *φ*_*i*_ = (*i* − 1)π/5, still for the purpose of cylindrical symmetry. Rewriting the polarization parameters as shown in Eq. (11), we have the engineered parameters $\beta_{i},\gamma_{i}^{\left(1\right)},\gamma_{i}^{\left(2\right)},$ and *A*_*i*_, with *A*_*i*_ confined to be greater than 0.05.

**Figure 3:**
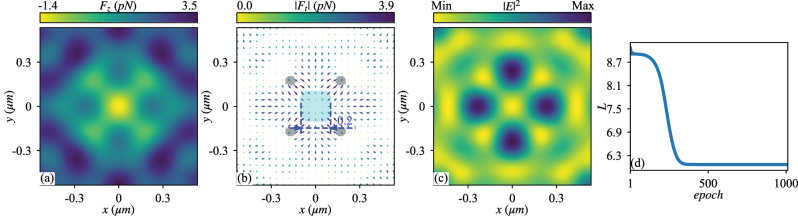
The same as Figure 2 except that the particle has a radius of *r*_*s*_ = 0.5*λ*_0_ and *ɛ*_*s*_ = 2.53 while the optimized structured light is composed of 10 plane waves with a diversity of polarization as presented in Table 1 in Supplementary Information and the first plane wave has *A*_1_ *E*_0_ = 8.68 × 10^5^ V/m. It produces a trapped area with negative longitudinal OF denoted by the central shaded area in panel (b). Although the dielectric particle is transversely trapped by the gradient OF, it is stabilized at the intensity minimum, contrast to the conventional case shown in Figure 2.

With the ADAM optimizer and back-propagation, we optimize the structured light, with learning rate equal to 0.001, to produce the desired OF. The optimized OFs, light intensity and change of loss function are shown in Figure 3. The engineered parameters of each plane wave optimized for the desired OF are shown in Table 1 in Supplementary Information.

Different from the previous case shown in Figures 2 and 3 implies that the particle is transversely trapped at light intensity minimum, a somewhat counter-intuitive phenomenon due to the effect of particle size. This is of no surprise. Because for particle of finite size, the gradient force is proportional to the gradient of light intensity that is a weighted average over the volume occupied by the particle, instead of the intensity exactly at particle center. In Figure 3, the particle centered at the origin actually occupies a volume with the largest averaged intensity and is thus trapped therein.

The optimized *p*_*i*_ and *q*_*i*_ in Table 1 imply *p*(*φ*_*i*_ + π) ≊ *p*(*φ*_*i*_) and *q*(*φ*_*i*_ + π) ≊ *q*(*φ*_*i*_), suggestive of the structured light propagating with a vortex factor *e* ^2*iφ*^. We have thus tried to generate a trapped area with pulling OF on such a particle using the Bessel beams of zero- and second-order, respectively. Both beams consist of the superimposed TM and transverse electric (TE) modes, with the relative amplitude and phase of the two modes optimized for our purpose. The optimal results are shown in Figure 8 in Supplementary Information. While the zero-order beam fails to produce a transversely trapped area for the pulling OF, the second-order beam, which carries orbital angular momentum, exhibits a vortex in the OF field, likely ruining the transverse confinement [7] of the particle to the area of pulling OF.

## 4 Conclusions

In this paper, we have demonstrated a back-propagation-based inverse design approach for devising a structured light field to achieve the predefined pattern of OF exerted on a particle of arbitrary size. The approach is built on reformulating the OF expression in a closed form in terms of only the elementary functions, avoiding the special functions within the conventional framework of GLMT. It thus permits the direct use of the back-propagation to analytically compute the gradients of the loss function with respect to the engineered parameters that govern the structured light field, without resorting to NNs. Besides facilitating the analytical calculation of the gradients, the NN is used also to expedite many physics simulations by its excellence in function approximation. Our algorithm is exact in the OF calculation, with the computing speed comparable to the trained NN. Then, with appropriately designed loss function, the ADAM optimizer is applied to optimize the engineered parameters, leading eventually to a structured light field that reproduce the predefined profile of the OF with reasonable accuracy. Some demonstration examples are presented to illustrate the approach. They are completed on a mobile computer with NVIDIA GeForce GTX 1060 (6G), indicating that it is not hardware demanding as an approach to attack the inverse design problem that usually needs large computation resources. In addition, the algorithm supports the adjustment of the training samples as well as the change of the number of waves that form the structured light, showing great flexibility. Also, our approach can be directly applied to reproduce the three-dimensional pre-designed patterns of OF for both transverse and longitudinal optical manipulation [65–67]. Work along this line is in progress.

Finally, our inverse design algorithm can benefit further from the ideas in the theory of NNs to improve its functionality. Here are two examples. (i) Regularization plays an important role in our optimization towards the appropriate structured light. Without regularization, the amplitude of the light field tends to increase, resulting in, sometimes, a much smaller OF in the training area than in other areas, or, in some other cases, leading to very large amplitude difference between different constituent plane waves. This is obvious not conducive to optical manipulation or experimental implementation of the designed structured light by spatial light modulators. This is reminiscent of the ‘overfitting’ [68] in NNs. (ii) In this paper, we have given the approximate wave vector directions before training. When doing the inverse design for new OF requirements, we can start with a larger number of constituent plane waves and pre-train without regularization. The plane waves with higher amplitude in the training usually suggest their more significant effect on producing the desired OF. We may imitate ‘pruning’ [69, 70] to remove those plane waves with low amplitude, then introduce the regularization, and restart the optimization with the approximate range of wave vectors adopted from the survived ones.

## Supplementary Material

Supplementary Material Details
