# Supplementary material for: Back-propagation-assisted inverse design of structured light fields for given profiles of optical force
Source: Nanophotonics. 2023 May 1;12(11):2019–27. doi: 10.1515/nanoph-2023-0101 (PMC11614335; doi:10.1515/nanoph-2023-0101)
Supplement: Supplementary file 1 — Supplementary Material Details [file j_nanoph-2023-0101_suppl_001.pdf]

This document provides supplementary information to “Back-propagation-assisted inverse design of structured light fields for given profiles of optical force”.

1 Explicit expressions for $Q^{(n)}_{l,ij}$, $R^{(n)}_{l,ij}$, and $N^{(n)}_{ij}$

$Q^{(1)\sim(2)}_{l,ij}$ and $R^{(1)\sim(7)}_{l,ij}$ in Eq. (3) are polynomials dependent on x_{ij}, where $x_{ij} = \boldsymbol{k}_i \cdot \boldsymbol{k}_j$ with $\boldsymbol{k}_i$ denoting the i-th wave vector. They are given by

$$
\begin{aligned}
Q^{(1)}_{l,\,ij} &= \sum_{m=1}^{l}{}^{(2)} m(2l+1-m)(2l+1-2m)P_{l-m}(x_{ij}),\\
Q^{(2)}_{l,\,ij} &= \sum_{m=2}^{l}{}^{(2)} m(2l+1-m)(2l+1-2m)P_{l-m}(x_{ij}),\\
R^{(1)}_{l,\,ij} &= \sum_{m=1}^{l}{}^{(2)} (m+1)(2l+2-m)(2l+1-2m)[2(m+1)l-(m^2-m-4)]P_{l-m}(x_{ij}),\\
R^{(2)}_{l,\,ij} &= \sum_{m=2}^{l}{}^{(2)} m(m+2)(2l+1-m)(2l+1-2m)(2l+3-m)P_{l-m}(x_{ij}),\\
R^{(3)}_{l,\,ij} &= \sum_{m=1}^{l}{}^{(2)} (m+1)(2l+2-m)(2l+1-2m)P_{l-m}(x_{ij}),\\
R^{(4)}_{l,\,ij} &= \sum_{m=2}^{l}{}^{(2)} (2l+1-m)(2l+1-2m)[2m^2l-m(m+1)(m-2)]P_{l-m}(x_{ij}),\\
R^{(5)}_{l,\,ij} &= \sum_{m=1}^{l}{}^{(2)} (m+1)(m-1)(2l-m)(2l+2-m)(2l+1-2m)P_{l-m}(x_{ij}),\\
R^{(6)}_{l,\,ij} &= \sum_{m=2}^{l}{}^{(2)} m(2l+1-m)(2l+1-2m)P_{l-m}(x_{ij}),\\
R^{(7)}_{l,\,ij} &= \sum_{m=1}^{l}{}^{(2)} (2l+1-2m)[2l^2-2(m-1)l+m^2-m]P_{l-m}(x_{ij}),
\end{aligned}
\tag{15}
$$

where $\sum_{m=1}^{l}{}^{(2)}$ and $\sum_{m=2}^{l}{}^{(2)}$ denote, respectively, the summation index m assuming odd and even positive integers satisfying $0 < m \le l$, and $P_l(\cdot)$ are the Legendre polynomials.

The coefficients $N_{ij}^{(1)\sim(12)}$ in Eq. (6) depend on θ_i, φ_i, θ_j, and φ_j,where θ_i and φ_i are polar angle and azimuthal angle of i-th wave vector $\boldsymbol{k}_i$, respectively. They read

$$
\begin{aligned}
N_{ij}^{(1)} &= C_{ij}^{(1)} W_{ij}^{(1)} \cos\theta_j \cos\varphi_j - C_{ij}^{(2)} W_{ij}^{(2)} \sin\varphi_j + C_{ij}^{(3)} \sin\theta_i \cos\varphi_j, \\
N_{ij}^{(2)} &= -C_{ij}^{(1)} W_{ij}^{(2)} \sin\varphi_i + C_{ij}^{(2)} W_{ij}^{(1)} \cos\theta_j \cos\varphi_j + C_{ij}^{(3)} \cos\varphi_i \sin\theta_j, \\
N_{ij}^{(3)} &= -C_{ij}^{(1)} W_{ij}^{(1)} \sin\varphi_j - C_{ij}^{(2)} W_{ij}^{(2)} \cos\theta_j \cos\varphi_j + C_{ij}^{(3)} (\sin\varphi_i \sin\theta_j - \cos\theta_j \sin\varphi_j) \sin\theta_i, \\
N_{ij}^{(4)} &= C_{ij}^{(1)} W_{ij}^{(2)} \cos\theta_j \cos\varphi_j + C_{ij}^{(2)} W_{ij}^{(1)} \sin\varphi_j, \\
N_{ij}^{(5)} &= C_{ij}^{(1)} W_{ij}^{(1)} \cos\theta_j \sin\varphi_j + C_{ij}^{(2)} W_{ij}^{(2)} \cos\varphi_j + C_{ij}^{(3)} \sin\theta_i \sin\varphi_j, \\
N_{ij}^{(6)} &= C_{ij}^{(1)} W_{ij}^{(2)} \cos\varphi_j + C_{ij}^{(2)} W_{ij}^{(1)} \cos\theta_j \sin\varphi_j + C_{ij}^{(3)} \sin\varphi_i \sin\theta_j, \\
N_{ij}^{(7)} &= C_{ij}^{(1)} W_{ij}^{(1)} \cos\varphi_j - C_{ij}^{(2)} W_{ij}^{(2)} \cos\theta_j \sin\varphi_j + C_{ij}^{(3)} (\cos\theta_j \cos\varphi_j - \cos\varphi_i \sin\theta_j) \sin\theta_i, \\
N_{ij}^{(8)} &= C_{ij}^{(1)} W_{ij}^{(2)} \cos\theta_j \sin\varphi_j - C_{ij}^{(2)} W_{ij}^{(1)} \cos\varphi_j, \\
N_{ij}^{(9)} &= -C_{ij}^{(1)} W_{ij}^{(1)} \sin\theta_j + C_{ij}^{(3)} (\cos\varphi_i \cos\varphi_j + \sin\varphi_i \sin\varphi_j) \sin\theta_i, \\
N_{ij}^{(10)} &= -C_{ij}^{(2)} W_{ij}^{(1)} \sin\theta_j + C_{ij}^{(3)} (\cos\varphi_i \cos\varphi_j + \sin\varphi_i \sin\varphi_j) \cos\theta_j, \\
N_{ij}^{(11)} &= C_{ij}^{(2)} W_{ij}^{(2)} \sin\theta_j + C_{ij}^{(3)} (\sin\varphi_i \cos\varphi_j - \cos\varphi_i \sin\varphi_j) \sin\theta_i \cos\theta_j, \\
N_{ij}^{(12)} &= -C_{ij}^{(1)} W_{ij}^{(2)} \sin\theta_j - C_{ij}^{(3)} (\sin\varphi_i \cos\varphi_j - \cos\varphi_i \sin\varphi_j),
\end{aligned}
\tag{16}
$$

with

$$
\begin{aligned}
W_{ij}^{(1)} &= -\cos\theta_i \cos\varphi_i \sin\theta_j \cos\varphi_j - \cos\theta_i \sin\varphi_i \sin\theta_j \sin\varphi_j + \sin\theta_i \cos\theta_j, \\
W_{ij}^{(2)} &= \sin\varphi_i \sin\theta_j \cos\varphi_j - \cos\varphi_i \sin\theta_j \sin\varphi_j,
\end{aligned}
\tag{17}
$$

and

$$
\begin{aligned}
C_{ij}^{(1)} =& \sum_l \big[u_l^{(1)} a_l Q_{l,ij}^{(1)} - u_l^{(1)} b_l Q_{l,ij}^{(2)} - u_l^{(2)} a_{l+1}^* a_l R_{l,ij}^{(1)} + u_l^{(2)} b_{l+1}^* b_l R_{l,ij}^{(2)} \\
& + (u_l^{(2)} a_{l+1} a_l^* + u_l^{(3)} a_l^* b_l) R_{l,ij}^{(4)} - (u_l^{(2)} b_{l+1} b_l^* + u_l^{(3)} a_l b_l^*) R_{l,ij}^{(5)} \big], \\
C_{ij}^{(2)} =& \sum_l \big[u_l^{(1)} b_l Q_{l,ij}^{(1)} - u_l^{(1)} a_l Q_{l,ij}^{(2)} - u_l^{(2)} b_{l+1}^* b_l R_{l,ij}^{(1)} + u_l^{(2)} a_{l+1}^* a_l R_{l,ij}^{(2)} \\
& + (u_l^{(2)} b_{l+1} b_l^* + u_l^{(3)} a_l b_l^*) R_{l,ij}^{(4)} - (u_l^{(2)} a_{l+1} a_l^* + u_l^{(3)} a_l^* b_l) R_{l,ij}^{(5)} \big], \\
C_{ij}^{(3)} =& \sum_l \big[-(u_l^{(1)} a_l + u_l^{(1)} b_l^*) Q_{l,ij}^{(1)} + (u_l^{(1)} b_l + u_l^{(1)} a_l^*) Q_{l,ij}^{(2)} - 4u_l^{(2)} (a_{l+1}^* a_l + b_{l+1} b_l^*) R_{l,ij}^{(3)} \\
& + (u_l^{(2)} a_{l+1}^* a_l + u_l^{(2)} b_{l+1} b_l^*) R_{l,ij}^{(1)} - (u_l^{(2)} b_{l+1}^* b_l + u_l^{(2)} a_{l+1} a_l^*) R_{l,ij}^{(2)} \\
& - \big(u_l^{(2)} (a_{l+1} a_l^* + b_{l+1}^* b_l) + 2u_l^{(3)} a_l^* b_l \big) R_{l,ij}^{(4)} + \big(u_l^{(2)} (b_{l+1} b_l^* + a_{l+1}^* a_l) + 2u_l^{(3)} a_l b_l^* \big) R_{l,ij}^{(5)} \\
& + 4\big(u_l^{(2)} (a_{l+1} a_l^* + b_{l+1}^* b_l) + u_l^{(3)} a_l^* b_l \big) R_{l,ij}^{(6)} + 4u_l^{(3)} a_l b_l^* R_{l,ij}^{(7)} \big],
\end{aligned}
\tag{18}
$$

with

$$
u_l^{(1)} = -\frac{\pi(2l+1)}{l(l+1)}, \quad u_l^{(2)} = -\frac{\pi}{2(l+1)^2}, \quad u_l^{(3)} = -\frac{\pi(2l+1)}{2l^2(l+1)^2}.
$$

Seeming rather complicated, the expressions involve only only polynomials and trigonometric functions concerning the engineered parameters, enabling a direct use of the back-propagation algorithm with currently available machine learning framework. We may also complete the complex calculations of some coefficients before training in many cases.

2 Additional numerical results for Section 3

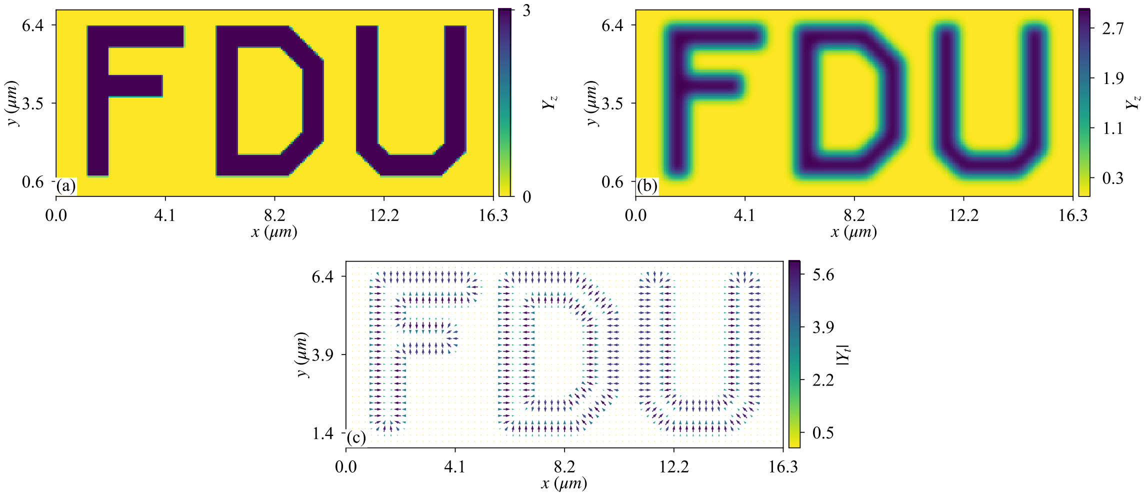

Fig. 4: (a) and (b) The prespecified pattern of the longitudinal optical force (OF) F_z before (a) and after (b) convolutions. (c) The pre-designed transverse trapping OF $\boldsymbol{Y}_t(x, y)$ with the arrows denoting the directions of $\boldsymbol{Y}_t(x, y)$, while their lengths and colors representing the magnitudes of $\boldsymbol{Y}_t(x, y)$.

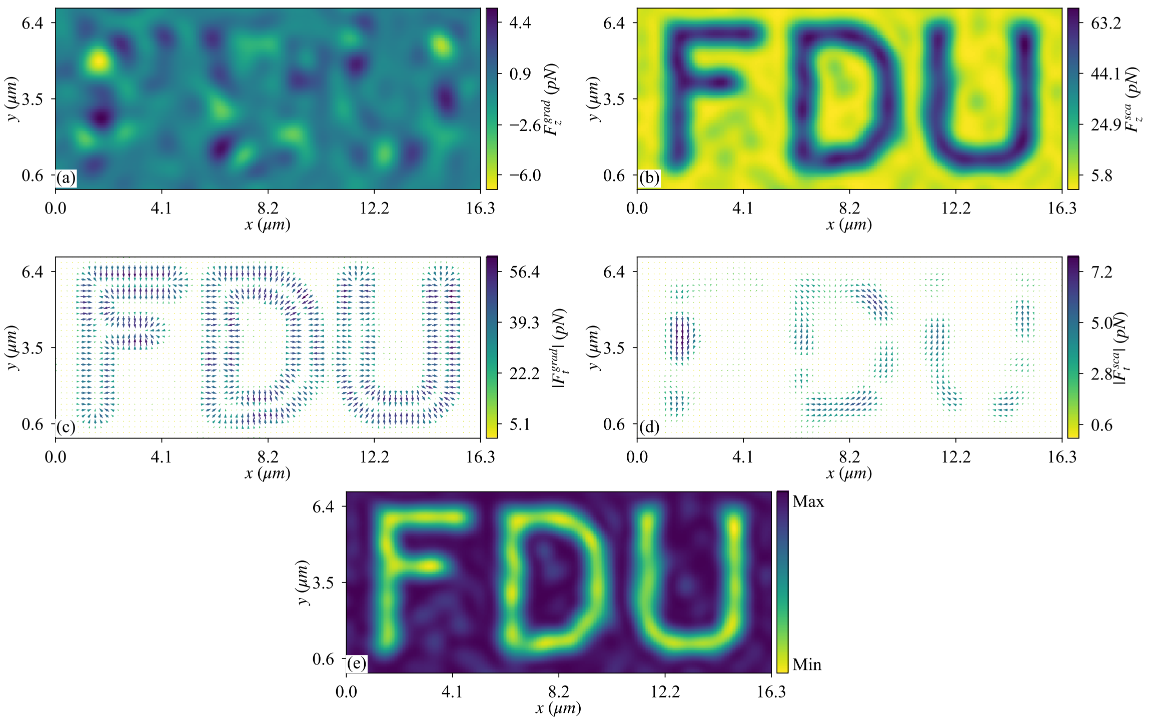

Fig. 5: (a) and (b): The calculated gradient (a) and scattering (b) parts of the longitudinal OF F_z when a particle is illuminated by an optimized structured light field. All parameters are the same as in Figure 1 in the main text. The scattering force dominates the longitudinal OF. (c) and (d): The same as panels (a) and (b) except for the transverse OF $\boldsymbol{F}_t$. The arrows denote the directions of $\boldsymbol{F}_t$, while their lengths and colors represent the magnitudes of $\boldsymbol{F}_t$. The particle is trapped basically by the gradient force. (e) The optical potential φ_t for the gradient part $\boldsymbol{F}_t^{\text{grad}}$ of the transverse OF. The optical potential is defined by $\boldsymbol{F}_t^{\text{grad}} = -\nabla\varphi_t$.

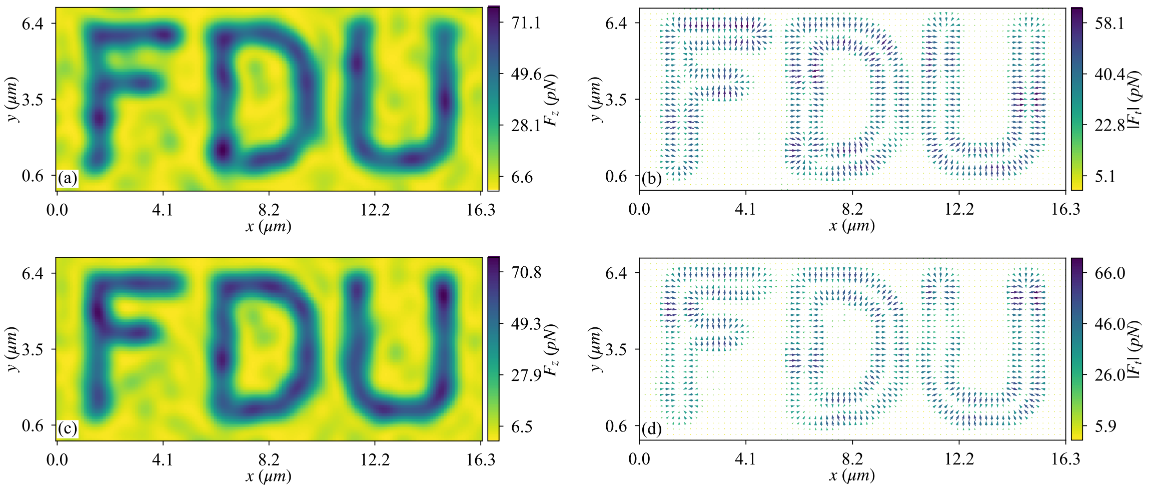

Fig. 6: The same as panels (a) and (b) in Figure 1 except that the OF are calculated at $z = 3\lambda$ (a) and (b) and $z = -3\lambda$ (c) and (d). The results indicate that the particle can be trapped by the designed structured light obtained in Figure 1 inside of the 'FDU' pattern within the range of $-3\lambda \lesssim z \lesssim 3\lambda$.

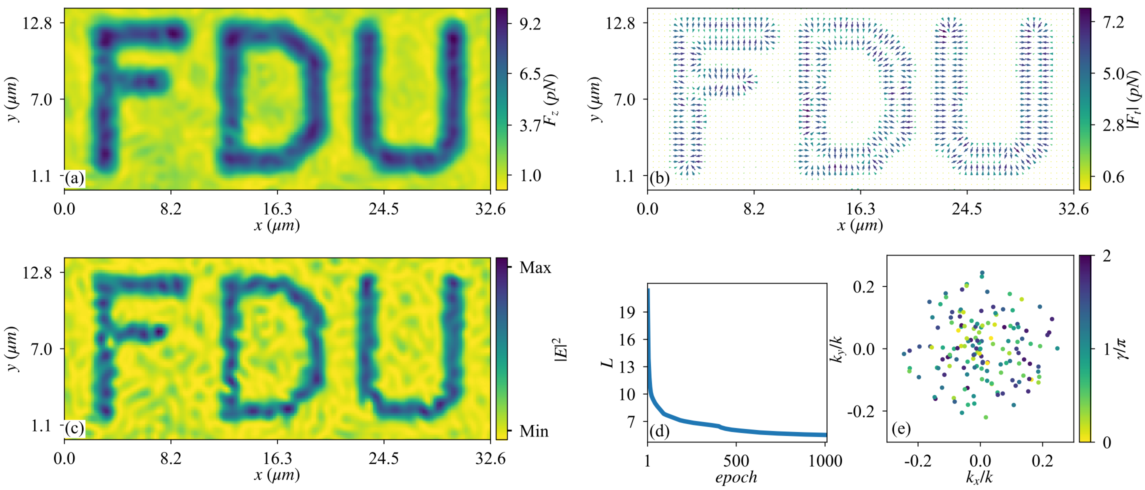

Fig. 7: The training is the same as Figure 1 except that the particle has a radius of $1.2\lambda_0$, training area is doubled in length and width with a resolution of 0.4λ, and the number of plane waves is $n_p = 150$ with each plane wave having $AE_0 = 8.68 \times 10^4$ V/m.

Tab. 1: The engineered parameters of the structured light for optical pulling in Figure 3.

φ_i	A_i	β_i	$\gamma_i^{(1)}$	$\gamma_i^{(2)}$	p_i	q_i
0	0.1973	0	0	–	0.197	0
0.2π	0.1792	0.41π	0.07π	1.21π	$0.047 + 0.011i$	$-0.138 - 0.104i$
0.4π	0.1756	0.20π	1.01π	1.16π	$-0.141 - 0.005i$	$-0.091 - 0.052i$
0.6π	0.1756	0.20π	1.01π	0.16π	$-0.141 - 0.005i$	$0.091 + 0.052i$
0.8π	0.1792	0.41π	0.07π	0.21π	$0.047 + 0.011i$	$0.138 + 0.104i$
π	0.1973	0	0	–	0.197	0
1.2π	0.1792	0.41π	0.07π	1.21π	$0.047 + 0.011i$	$-0.138 - 0.104i$
1.4π	0.1756	0.20π	1.01π	1.16π	$-0.141 - 0.005i$	$-0.091 - 0.052i$
1.6π	0.1756	0.20π	1.01π	0.16π	$-0.141 - 0.005i$	$0.091 + 0.052i$
1.8π	0.1792	0.41π	0.07π	0.21π	$0.047 + 0.011i$	$0.138 + 0.104i$

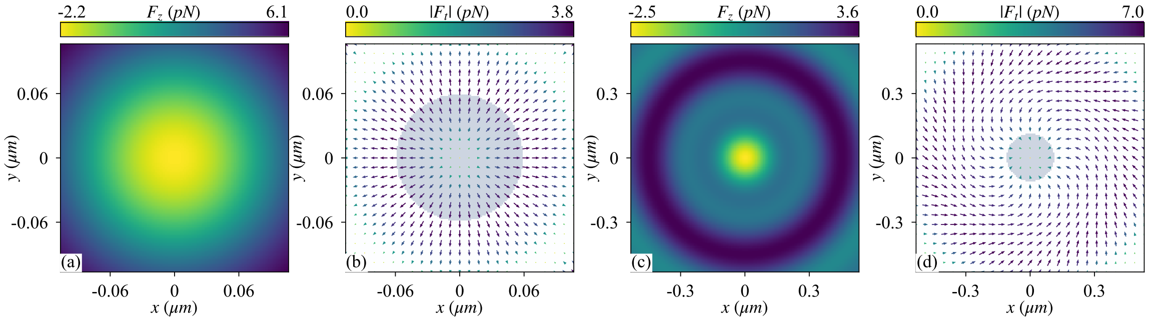

Fig. 8: The calculated longitudinal OF F_z (a, c) and transverse trapping OF $\boldsymbol{F}_t$ (b, d) exerted on a particle of $r_s = 0.5\lambda_0$ and $\varepsilon_s = 2.53$ illuminated by a Bessel beam with zero-order (a, b) and second-order (c, d). E_0 at the first intensity maximum of the beam is set as 6.1×10^6 V/m and 4.5×10^6 V/m for the zero- and second- order beams, respectively. Bessel beams optimized with respect to the relative amplitude and phase of the TM and TE modes for producing a transverse trapped area with a longitudinal pulling OF. In panels (b) and (d), the shaded area represent the area with pulling OF, the arrows denote the directions of $\boldsymbol{F}_t$, while their lengths and colors denote the magnitudes of $\boldsymbol{F}_t$. The zero-order beam fails to serve our purpose since the particle cannot be trapped in the negative OF area, while the second-order beam generates a force field with a vortex, adding considerably to the difficulty of constraining the particle inside of the negative OF area.
